# Editorial: Functional Genomics of Transcriptional Regulation in Pathogenic Fungi

**DOI:** 10.3389/fcimb.2022.928440

**Published:** 2022-06-01

**Authors:** Bernard Turcotte

**Affiliations:** Department of Medicine, McGill University Health Centre, Montréal, QC, Canada

**Keywords:** pathogenic fungi, functional genomics, *Candida albicans*, *Candida glabrata*, *Microsporum canis*, transcription factors

Approximately one hundred thousand fungal species have been described and some studies estimate that there are over five million (Blackwell, 2011). Many fungi play a positive role in various ecosystems, such as their symbiotic association with plants while others have been successfully used in the biotechnology field, as exemplified by the baker’s yeast *Saccharomyces cerevisiae* and *Komagataella phaffii* (*Pichia pastoris*). However, a number of fungal species constitute a major threat to plants and animals, including humans (Fisher et al., 2012). In humans, fungi are responsible for 1.5 million deaths each year with *Aspergillus*, *Candida*, and *Cryptococcus* species being the fungal pathogens generating the majority of cases of serious fungal disease. *Candida albicans* is the principal cause of invasive infections with *C. glabrata* ranking second; other fungi play less lethal roles, such as *Microsporum canis* which is a common skin fungus. In this series of publications, the authors describe the role of a number of transcriptional regulators involved in controlling diverse processes in a variety of pathogenic fungi.

*C. albicans* is a typically commensal organism that inhabits the mucosal linings of warm-blooded animals, but as stated above, it is also the major culprit in human fungal infections. *C. albicans* is a severe and persistent opportunistic pathogen of immunocompromised individuals. Rogriguez et al. reviewed gene networks involved in the regulation of developmental processes in *C. albicans*. The authors provide a detailed assessment of the factors that form the basis of transcriptional circuits involved in controlling three central developmental processes. (1) association of virulence of *C. albicans* with its ability to switch from yeast to hyphae (and vice-versa), (2) ability of *C. albicans* to switch from white to opaque forms (a process important for its parasexual cycle and (3) the formation of biofilms containing a protective extra-cellular matrix, a structure providing resistance to antifungal treatment. The authors also discuss the interconnection among these circuits.

In *C. albicans*, the role of many transcription factors has been uncovered using transcriptomics and genome-wide location analyses. However, the majority of these studies have been performed under normoxic conditions even though hypoxia is a condition frequently encountered in the human host and is a major signal for filamentous growth. Henry et al. were interested in better characterizing the link between hypoxia and growth in a filamentous form. A genetic screen with deletion mutants identified a number of factors involved in this process, including the transcriptional regulators Ahr1 and Tye7 that were known to regulate genes involved in glycolysis and adhesion, respectively. The authors show that Ahr1 and Tye7 act as negative regulators of filamentation under hypoxia. Genetic interaction analysis showed that Ahr1 and Tye7 down-regulate filamentation under hypoxia through the Efg1 and Ras1/Cyr1 pathways.

Liboro et al. had previously shown that *C. albicans* Yck2 (Yeast Casein Kinase) controls the switch from yeast to hyphae. In this study (Liboro et al.), they determine the transcriptome and the metabolome of a strain lacking *YCK2*. Results show that the transcriptome of a Δ*yck2* strain resembles that of *C. albicans* responding to engulfment by macrophages. For example, genes involved in the glyoxylate cycle, the response to oxidative stress, beta-oxidation, and arginine biosynthesis are upregulated in a strain lacking *YCK2*. Metabolome analysis showed higher levels of methyl citrate cycle intermediates. Thus, Yck2 plays a role in carbon metabolism and morphogenesis.

SAGA is a multi-protein complex conserved from budding yeast to humans and is involved in regulating multiple processes including modification of chromatin structure and regulation of gene expression. SAGA has two enzymatic activities: it acts as a histone acetyltransferase and a histone deubiquitinase. Rashid et al. studied this complex in *C. albicans*. They used deletion or conditional mutants targeting the five SAGA modules: the two enzymatic modules (Ngg1, Ubp8), the recruitment module (Tra1) involved in interacting with transcriptional activators, the core structural module (Spt7) and a subunit involved in the recruitment of the TATA binding protein (TBP) to target promoters (Spt8). The various mutants were tested for growth, morphogenesis, invasiveness, biofilm formation and environmental stresses (e.g. oxidative stress). This led to the identification of many phenotypes. For example, strains lacking *SPT7* or *SPT8* show an increased filamentation and increased invasiveness as compared to a wild-type strain. In contrast, Δ*ngg1* and Δ*ubp8* strains do not form hyphae and are non-invasive. This study provides a better understanding of the role of the SAGA complex in the response to environmental cues.

Delaveau et al. studied the highly conserved CCAAT-binding complex (CBC) in *C. glabrata*. CBC is a trimer constitutively bound to DNA and its association with either Yap5 or Hap4 allows regulation of different sets of genes. Yap5 is involved in controlling respiratory genes while Hap4 controls response to toxic iron concentrations. These observations raise the question about how these two pathways can be independent of each other and how their interference is prevented. To answer this question, Delaveau et al. used different approaches including expression profiling and ChIP analysis. Results show that regulation of iron tolerance by Yap5 is dependent not only on its interaction with CBC but also with a Yap-response element that needs to be in close proximity of a CBC DNA binding site. In addition, Yap5 competes with Hap4 for binding to CBC at iron tolerance genes. These results show how it is possible for two transcription factors to differentially regulate two pathways sharing a common DNA binding complex.

Dai et al. studied the effect of zinc deficiency in *M. canis*. RNA-seq analysis was performed under zinc-limiting conditions. Expression of the ZafA gene encoding a transcriptional activator, was increased with zinc deficiency. A strain lacking ZafA showed reductions in zinc adsorption, in biodegradation of hair, and in pathogenicity. The authors suggest that ZafA could be used as a potential drug target.

In summary, in this series of publications, genome-wide approaches have been used to perform functional studies aimed at unraveling the roles of transcription factors in pathogenic fungi. These studies provide useful information about transcriptional regulation for response to environmental cues as they organisms have developed transcription strategies to quickly adapt to changing niches. These studies may be helpful to identify new targets for designing new antifungal drugs.

## Author Contributions

The author confirms being the sole contributor of this work and has approved it for publication.

## Funding

BT was supported by a grant from the Fonds de Recherche du Québec- Nature et Technologie. BT is grateful to Dr. Malcolm Whiteway (Concordia University) for critical review of the editorial.

## Conflict of Interest

The author declares that the research was conducted in the absence of any commercial or financial relationships that could be construed as a potential conflict of interest.

## Publisher’s Note

All claims expressed in this article are solely those of the authors and do not necessarily represent those of their affiliated organizations, or those of the publisher, the editors and the reviewers. Any product that may be evaluated in this article, or claim that may be made by its manufacturer, is not guaranteed or endorsed by the publisher.
